# Supplementary material for: Impact of childhood traumatic brain injury on fitness for service class, length of service period, and cognitive performance during military service in Finland from 1998 to 2018: A retrospective register-based nationwide cohort study
Source: PLoS One. 2024 May 20;19(5):e0303851. doi: 10.1371/journal.pone.0303851 (PMC11104597; doi:10.1371/journal.pone.0303851)
Supplement: S1 Appendix — (DOCX) [file pone.0303851.s001.docx]

**S1 Appendix.**

**ICD-10 Diagnose codes and operation codes used in the study.**

S1 Table. Diagnose codes used to identify pTBI (ICD-10 diagnostic code system).

| Diagnose code | Definition |
| --- | --- |
| S06.0 | Concussion |
| S06.1 | Traumatic cerebral edema |
| S06.2 | Diffuse traumatic brain injury |
| S06.3 | Focal traumatic brain injury |
| S06.4 | Epidural hemorrhage |
| S06.5 | Traumatic subdural hemorrhage |
| S06.6 | Traumatic subarachnoid hemorrhage |
| S06.7 | Intracranial injury and prolonged concussion |
| S06.8 | Other intracranial injury |
| S06.9 | Unspecified intracranial injury |

S2 Table. Diagnose codes used to identify the reference group of wrist and ankle fractures

| Diagnose code | Definition |
| --- | --- |
| S52.0 | Fracture of upper end of ulna |
| S52.1 | Fracture of upper end of radius |
| S52.2 | Fracture of shaft of ulna |
| S52.3 | Fracture of shaft of radius |
| S52.4 | Fracture of shaft of radius and ulna |
| S52.5 | Fracture of lower end of radius |
| S52.6 | Fracture of lower end of radius and ulna |
| S52.7 | Multiple fractures of ulna |
| S52.8 | Fracture of other part of ulna |
| S52.9 | Unspecified fracture of forearm |
| S82.0 | Fracture of patella |
| S82.1 | Fracture of upper end of tibia |
| S82.2 | Fracture of shaft of tibia |
| S82.3 | Fracture of lower end of tibia |
| S82.4 | Fracture of shaft of fibula |
| S82.5 | Fracture of medial malleolus |
| S82.6 | Fracture of lateral malleolus |
| S82.7 | Multiple fractures of tibia or fibula |
| S82.8 | Other fractures of lower leg |
| S82.9 | Unspecified fracture of lower leg |

S3 Table. Neurosurgery codes (NCSP Finnish version).

| Neurosurgery code | Definition |
| --- | --- |
| AAD00 | Evacuation of epidural hematoma |
| AAD05 | Evacuation of acute subdural hematoma |
| AAD15 | Evacuation of traumatic intracerebral hematoma |
| AAF00 | Ventriculostomy |
| AAK80 | Partial excision of skull cap for relief of acute cerebral edema |
