# Supplementary material for: Impact of childhood traumatic brain injury on fitness for service class, length of service period, and cognitive performance during military service in Finland from 1998 to 2018: A retrospective register-based nationwide cohort study
Source: PLoS One. 2024 May 20;19(5):e0303851. doi: 10.1371/journal.pone.0303851 (PMC11104597; doi:10.1371/journal.pone.0303851)
Supplement: Attachment — Submitted filename: Response to reviewers.docx [file pone.0303851.s002.docx]

**Author responses to reviewers’ comments**

Dear Editor:

We would like to take this opportunity to thank the Reviewer(s) for their constructive criticism concerning our manuscript. We have revised the manuscript in accordance with the comments and suggestions received from the Reviewer. As a result, we feel that the quality of our manuscript has been greatly improved.

Please find below a specific list of the comments received from Reviewer followed by our responses in bold font. In addition, we have submitted a revised version of the manuscript with the changes highlighted in red as well as revised version without highlighted changes.

DETAILED COMMENTS:

1)

It is not clear why this research is needed. How will it impact future fit for service guidelines or medical recommendations for paediatric patients with these injuries?

***Author response: Thank you for this comment. We agree that this information was missing. We have now assessed this thoroughly in the manuscript. Please see, Introduction: Paragraph 4. and Discussion: Paragraph 5.***

2)

Please be clear in the methods - if conscription guidelines explicitly exclude people with pTBI.

***Author response: The reviewer 1 is correct. The Methods lacked detailed information how the service class is determined. We have now added paragraph to explain the reasoning behind the classification Simply, any form of a TBI is not an automatic exclusion from the service, everyone and every case is evaluated individually. Please, see Methods: Paragraph 4 and Discussion: Paragraph 4.***

3)

The discussion states: “there are numerous post-injury disorders and emotional disorders that are acceptable reasons for conscripts to receive an exemption from conscription or military service” later it goes on to say, “major operations are part of the exclusion criteria in the military handbook” Does this mean that recruits lie or are unaware of their past TBI history / major operation as a child? Does this bias the sample who make it into the service and who will eventually finish service? Is the research trying to elucidate this?

***Author response: Thank you for this comment. Please, see above. We have explained the exclusion criteria in a more detailed fashion.***

4)

Please include a table with the OR – I would suggest adding them to Table 1 and Table 2 respectively.

***Author response: We have now added all the ORs to tables as suggested. For the tables to be as clear as possible, we have created a new table for ORs regarding service length (Table 3). Previous Table 3 has now been moved to be Table 4 Please, see Results: Table 1, 3 and 4.***

5)

I note there was no matching between groups, but the number of females was surprisingly similar in both groups. Can the authors comment on incidence rates of TBI being similar to those of fractures? It is possible that randomly the groups are the same size, but it just really jumped out at me.

***Author response: Thank you for pointing this out. We were surprised as well. It is completely coincidental that the groups were almost exactly same size.***

6)

Age is identified in Figure 1 as the main (only) confounder of the association, but it is not adjusted for in the logistic regression. Please provide rationale.

***Author response: Thank you for this comment. It was indented to run all analyses with age adjustment. This statement has now been added to the Methods section. We also checked the logistic regression analyses and simply by honest mistake there were few analyses done without age adjustment. New analyses were performed, and results are now updated entirely regarding the ORs. There were only minor differences, and this error did not affect the final interpretation of the results. Please, see Methods: Paragraph 6, Results: Tables 1 and 3.***

7)

What is the sample size for the cognitive impairment test – if it is given 6-8 weeks after enrolment and the authors suggest some people might drop out by then ; the sample size might be smaller for this analysis.

***Author response: We agree with the Reviewer. Sample size has now been added to Table 1 and Results. Indeed, there were some individuals who completed military service, but had missing data from the cognitive b-test. We have added this as limitation. Please, see Results: Paragraph 5 and Table 1, Discussion: Paragraph 6.***

8)

I think it would be valuable to present b-test score by length of service –larger differences between groups might be observed.

***Author response: As you can see from the table below, conscripts with longer service length have better results in this test. Differences between groups are not larger. The test is done before anyone knows how long they will be serving and logically people with better test results will be selected more often to the longer service periods. This is why we chose to report the results in one group. The fact that pTBI group has lower average score is the most important independent factor and the effects are seen in the service length between groups. In addition, we have now calculated P-values for the b-test results using Welch two sample t-test. Please, see Results: Table 1.***

| Length of service | pTBI group | Reference group | Difference | pTBI group | Reference group | Difference |
| --- | --- | --- | --- | --- | --- | --- |
|  | Men | Men |  | Women | Women |  |
| 6 months | 4.28 | 4.40 | 0.12 CI 0.05-0.20 | 5.50 | 5.43 | 0.07 CI -2.36-2.50 |
| 9 months | 4.68 | 4.81 | 0.12 CI -0.00-0.25 | 4.74 | 4.48 | 0.25 CI -1.19-0.68 |
| 12 months | 5.34 | 5.50 | 0.16 CI 0.09-0.23 | 5.31 | 5.60 | 0.29 CI -0.21-0.80 |
| All | 4.47 | 4.89 | 0.15 CI 0.10-0.20 | 5.17 | 5.29 | 0.12 CI -0.28-0.52 |

9)

What difference in the b-test score would be clinically meaningful? Or in the military what cut off are used to determine final posting.

***Author response: B-test scores from 0 to 3 usually indicate rejection from more demanding 9 to 12-month duties. Please, see Methods: Paragraph 5.***

ABSTRACT:

1)

Reference category includes a range of lower limb fractures wider than just ankle and wrist.

***Author response: It is true. There also were shin fractures and fractures of patella. We have corrected this in the manuscript. Please, see Abstract: Paragraph 2 and Methods: Paragraph 2.***
